# Potato peel waste fermentation by *Rhizopus oryzae* to produce lactic acid and ethanol

**DOI:** 10.1002/fsn3.3670

**Published:** 2023-09-07

**Authors:** Gülsüm Ebru Ozer Uyar, Basar Uyar

**Affiliations:** ^1^ Department of Plant Protection Kocaeli University Kocaeli Turkey; ^2^ Department of Chemical Engineering Kocaeli University Kocaeli Turkey

**Keywords:** ethanol production, lactic acid production, particle size, potato peel waste, *Rhizopus oryzae*

## Abstract

Potato peel waste (PPW), a zero‐value by‐product generated from potato processing, is a promising fermentation substrate due to its large quantity of starch, nonstarch polysaccharides, lignin, protein, and lipid. *Rhizopus oryzae* is a filamentous fungus that is mainly known as a lactic acid producer and can ferment various agro‐wastes. This study aimed to use *R. oryzae* for the fermentation of PPW. A series of batch fermentations were conducted to investigate the effects of different PPW loading rates (2%–8%) and particle sizes (0–4 mm). Under an initial PPW loading rate of 8% and particle size of 1–2 mm, the maximum ethanol (18.83 g/L) and lactic acid (3.14 g/L) concentrations, the highest ethanol (9.41 g/L·day) and lactic acid (1.89 g/L·day) average production rates were obtained. Under these conditions, the yield of ethanol and lactic acid was 0.235 g/gPPW and 0.039 g/gPPW, respectively. *R. oryzae* was shown to utilize PPW as a substrate to produce value‐added bioproducts such as ethanol (major product) and lactic acid.

## Graphical abstract

*Rhizopus oryzae* is able to utilize potato peel waste to produce DP4+, glucose, lactic acid, and ethanol. Potato peel waste particle size of 1–2 mm and loading rate of 8% were optimal for fermentation. PPW size of less than 0.125 mm and loading rate of more than 8% are not suitable due to high viscosity. 18.83 g/L of ethanol was produced at a rate of 9.41 g/L day with a yield of 0.235 g/gPPW. 3.14 g/L of lactic acid was produced at a rate of 1.89 g/L day with a yield of 0.039 g/gPPW.

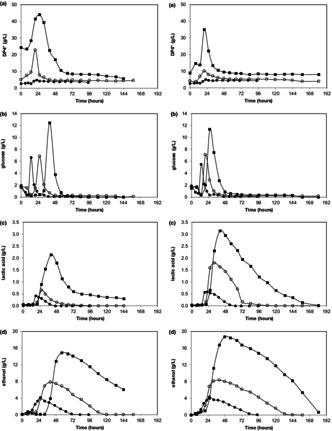

## 1 INTRODUCTION

*Rhizopus oryzae* (*Rhizopus arrhizus*) is one of the most economically important members of the zygomycete group of fungi. It has long been used for enzyme production (e.g., glucoamylase and lipase), organic acid synthesis (mainly lactic acid), and various fermented food applications (oriental foods and alcoholic beverages) (Ghosh & Ray, 2014).

Potato peel waste (PPW) is the main and zero‐value waste from potato processing plants, generating about 8% of the waste by weight. The problem of the disposal or management of PPW sustainably causes considerable concern to the potato industries in Europe and is ‐becoming a major issue in the USA, thus implying the need to identify an integrated, environmentally friendly solution. It is anticipated that around 8000 kilotons of PPW might be generated in 2030, with related greenhouse gas emissions of 5 million tons of CO_2_ equivalent (Khanal et al., 2023). The huge quantity of PPW generated annually also draws interest to the innovative and value‐added conversion of this carbon‐rich waste to value‐added by‐products with microorganisms in fermenters; notably to biohydrogen (Cao et al., 2022; Javed et al., 2019), biogas (Javed et al., 2019; Wu, 2016), biobutanol (Kamboj & Ms, 2021), bioethanol (Awogbemi et al., 2022), lactic acid (Arapoglou et al., 2010; Javed et al., 2019; Liang & McDonald, 2015; Wu, 2016), and enzymes (Khanal et al., 2023).

PPW has a high moisture content (80%–90%). The chemical composition of the dry matter reported in the literature differs greatly; however, it is certain that PPW has a high carbohydrate content (mainly starch followed by dietary fiber such as cellulose/hemicellulose/lignin), which makes it a good basis for fermentation. Overall protein and lipid contents are generally low (Arapoglou et al., 2010; Awogbemi et al., 2022; Barampouti et al., 2023; Javed et al., 2019; Khanal et al., 2023; Liang & McDonald, 2015; Sepelev & Galoburda, 2015). When compared to lignocellulosic substrates, which pose an additional challenge due to their recalcitrant structure and the difficult conversion of cellulose to fermentable sugars, PPW is a promising feedstock for biofuel production due to its high content in polysaccharides rich in starch. Also, its significant content of other nutrients, such as protein and salts, ensures the required nutritional needs for effective fermentation metabolism, eliminating the need for supplementation (Rodríguez‐Martínez et al., 2023).

*R. oryzae* was known to secrete a large number of carbohydrate‐digesting enzymes including amylases, cellulases, and hemicellulases, which makes it a candidate microorganism for exploiting PPW fermentation. The cellulase system in *R. oryzae* comprises two hydrolytic enzymes: extracellular endoglucanase and exoglucanase, and thus cellulosic wastes could be easily and rapidly converted into glucose without the requirement of alkali or acid pretreatments. Xylanase is an enzyme that catalyzes the hydrolysis of 1,4‐beta‐d‐xylosidic linkages in xylans that are constituents of hemicellulose and was shown to be readily produced by *R. oryzae* from different xylan‐containing agricultural by‐products. Finally, *R. oryzae* also possesses a starch‐breaking ability, it has been shown to produce extracellular isoamylase and glucoamylase to saccharify starch from potato, tamarind, tapioca, and oat (Ghosh & Ray, 2014).

PPW needs to be dried and grounded before being used as a substrate in a fermentation process. Here, one of the substrate‐related process parameters is the PPW particle size. As the particle size decreases, the surface area available for the hydrolysis reaction increases, which can lead to a higher production rate and yield (Meenakshi & Kumaresan, 2014; Yang et al., 2020). Grinding process was also shown to increase soluble dietary fiber composition and hydration characteristics of PPW (Yang et al., 2020). The solids loading rate is another important parameter associated with the effective fermentation of PPW, it has been previously shown to significantly affect lactic acid, acetic acid, and ethanol yields in an undefined mixed microbial culture (Liang et al., 2015).

Available studies in the literature either focus on the utilization of other agro‐wastes by *R. oryzae* or on the utilization of PPW by other microorganisms. This study intends to fill this gap, by addressing the use of PPW, which is a no‐value material, to obtain lactic acid and ethanol via fermentation by *R. oryzae*. In this context, the effects of loading rate and particle size of PPW on fermentation were investigated. The intermediary and final products obtained were defined and quantified. Rate and yield values were calculated to compare and assess the performance of the bioprocess.

## 2 MATERIALS AND METHODS

### 2.1 Microorganism and growth conditions

In this study, *R. oryzae* (ATCC 9363) was used. It was sporulated on streaked agar plates containing potato dextrose agar for 4–5 days at 30°C. After sporulation, the plates were stored at 4°C until the preparation of spore suspension. The spore suspension was obtained by washing the agar plates containing sporulated fungus with sterile water. Spore concentration in the suspension was determined by counting the spores on a hemocytometer.

For the fermentation study, 250‐mL Erlenmeyer flasks containing 100 mL of medium were used. The liquid medium is composed of (all w/v) 0.2% glucose, 0.2% (NH_4_)_2_SO_4_, 0.065% KH_2_PO_4_, 0.025% MgSO_4_·7H_2_O, 0.005% ZnSO_4_·7H_2_O, and PPW.

PPW samples were collected from the household disposals and kept frozen (at −20°C) until used. After thawing, PPW biomass was dried in an incubator at 75°C until it reached a constant weight. The dried biomass was grounded in a blender to a granular form and sieved to separate into size fractions: smaller than 0.125, 0.125–0.250, 0.250–0.500, 0.500–1.000, 1.000–2.000, and 2.000–4.000 (all in mm).

These six PPW size fractions were added to nutritional media at a loading rate of 2% in order to prepare the medium for the PPW particle size research and to prepare the nutritional media for the PPW loading rate investigation. The sizes (0.125–0.250 mm) and (1–2 mm) of PPW were added at loading rates of 2%, 4%, and 8%.

Flasks were inoculated with spore suspension to a final concentration of 10^5^ spores/mL. After the inoculation, the cultures were incubated in a shaker incubator at 35°C and 150 rpm. The total incubation time was 96 h for the PPW particle size study and 144 or 192 h (depending on particle size) for the PPW loading rate study.

### 2.2 Analytical methods

Glucose, DP4+ (high saccharides containing four or more glucose), lactic acid, and ethanol concentrations were determined by HPLC (Agilent 1200). The HPLC method is based on a normal chromatography procedure, using a Phenomenex Rezex Cal organic acid column, the size of which is 300 × 7.8 mm, and a refractive index detector. Eluent was 5 mM H_2_SO_4_ flowing at 0.6 mL/min. The volume of injection for both standard solutions and sample extracts was 20 μL.

### 2.3 Calculations of bioprocess parameters

Production rates were calculated in terms of g_product_/L_reactor_·day.

Yield (of lactic acid or ethanol) on a substrate is defined as the amount of the metabolite obtained per unit of PPW used and given in Equation (1):

(1) $$Y=\left(C–C_{0}\right)\cdot V/M\cdot 100\%$$

where *Y* is the yield (g/g), *C* is the actual concentration (g/L), *C*_0_ is the initial concentration (g/L), *V* is the bioreactor working volume (L), and *M* is the initial mass of PPW in the bioreactor (g).

Sugar recovery was defined as the efficiency of the microorganism to convert the starch available in the PPW to glucose. It is calculated as the ratio between the mass of glucose in the hydrolysate to the total mass of the starch in the PPW and expressed as a percentage (%).

A particularly useful parameter for characterizing ethanol and lactic acid production is substrate conversion efficiency, which is a measure of how much of the substrate has been utilized for ethanol and lactic acid production rather than growth, cell maintenance, or alternative biosynthesis. It was determined as the ratio of the mass of ethanol and lactic acid that has actually been produced per mass of ethanol and lactic acid expected through stoichiometric conversion of a substrate according to the hypothetical reactions of lactic acid and ethanol fermentations and expressed as a percentage (%).

### 2.4 Data analyses

The data reported are the average of three measurements.

First‐order (linear) regression analysis was performed to show any relation between the tested variables (independent variables such as particle size and particle loading rate) and the outcome (dependent variables such as produced amounts, average production rates and yields of ethanol, and lactic acid). The *R* ^2^ value, which quantifies the dispersion of distribution from the mean, was used as a measure of the goodness of fit.

Comparison of data sets obtained was made by one‐way analysis of variance (ANOVA) and Tukey's test. A difference was considered statistically significant if *p* < .05.

Calculations and regression analysis were made in MS Excel. For statistical analyses, Analysis ToolPak Add‐In was used.

## 3 RESULTS

### 3.1 Effect of particle size

**FIGURE 1:**
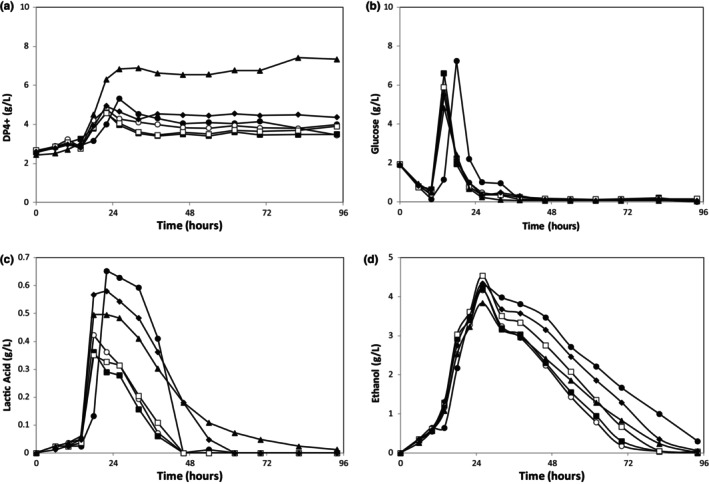
Time‐dependent concentration profiles of DP4+ (a), glucose (b), lactic acid (c), and ethanol (d) concentrations for different PPW sizes (●: <0.125, ○: 0.125–0.250, ■: 0.250–0.500, □: 0.500–1.000, ♦: 1.000–2.000, ▲: 2.000–4.000, all in mm).

In order to investigate how the bioprocess was affected by PPW particle size in the nutrient media, grounded PPW separated into six fractions in size (<0.125, 0.125–0.250, 0.250–0.500, 0.500–1.000, 1.000–2.000, and 2.000–4.000, all in mm) was tested. Nutrient media were prepared by using these PPW as the main substrate at a loading rate of 2%. In media prepared with a PPW particle size smaller than 0.125, some gelling was observed which resulted in a more viscous media. No such effect was observed for other PPW size ranges. The fermentations were carried out and DP4+, glucose, lactic acid, and ethanol concentrations were determined throughout the runs (Figure 1). Based on these data, sugar recovery, substrate conversion efficiency, produced amounts, average production rates, and yields of both ethanol and lactic acid were calculated and are tabulated in Table 1.

**TABLE 1:** Effect of the PPW particle size on the performance of the fermentations.

PPW particle size (mm)	Sugar recovery (%)	Substrate conversion efficiency (%)	Ethanol				Lactic acid			
			Time of peak production (h)	Maximum amount produced (g/L)	Average production rate (g/L day)	Yield (g/g_PPW_)	Time of peak production (h)	Maximum amount produced (g/L)	Average production rate (g/L day)	Yield (g/g_PPW_)
<0.125	72.3 ± 3.5	45.1 ± 1.9	26	4.28 ± 0.17	3.95 ± 0.15	0.214 ± 0.009	22	0.65 ± 0.04	0.71 ± 0.04	0.033 ± 0.002
0.125–0.250	66.0 ± 2.0	43.1 ± 1.5	26	4.19 ± 0.14	3.87 ± 0.13	0.209 ± 0.007	18	0.42 ± 0.02	0.56 ± 0.03	0.021 ± 0.001
0.250–0.500	66.1 ± 2.7	43.1 ± 1.9	26	4.22 ± 0.18	3.90 ± 0.17	0.211 ± 0.009	18	0.36 ± 0.03	0.48 ± 0.04	0.018 ± 0.001
0.500–1.000	59.2 ± 3.5	46.4 ± 1.5	26	4.56 ± 0.14	4.21 ± 0.13	0.228 ± 0.007	18	0.35 ± 0.02	0.47 ± 0.03	0.018 ± 0.001
1.000–2.000	54.7 ± 2.4	45.6 ± 2.0	26	4.36 ± 0.22	4.03 ± 0.20	0.218 ± 0.011	22	0.58 ± 0.04	0.63 ± 0.04	0.029 ± 0.002
2.000–4.000	48.3 ± 1.1	40.3 ± 1.4	26	3.86 ± 0.13	3.56 ± 0.13	0.193 ± 0.007	18	0.50 ± 0.03	0.66 ± 0.04	0.025 ± 0.002

*R. oryzae* utilized PPW as a substrate in all cases studied to produce glucose, DP4+, ethanol, and lactic acid.

It can be clearly seen in Figure 1 that the fungi show a distinct pattern of substrate utilization and consume back the metabolites they produced; all concentrations peak at the early stages of the fermentation and then decrease steadily. The consumption rates can be used as an indicator of the substrate preference order of the microorganism. Figure 1b shows that *R. oryzae* consumed the initial glucose available in the nutrient media within the first 10 h, and after this was depleted, it started to degrade the starch available in the PPW, as suggested by an increase in glucose and DP4+ concentrations (Figure 1a,b), followed by lactic acid and ethanol fermentations (Figure 1c,d). After the depletion of glucose, lactic acid, and ethanol consumption started and lasted until the end of the runs. Ethanol production was much higher compared to lactic acid production, for all cases.

Regression analysis did not show any meaningful correlation between PPW particle size and the produced amounts, average production rates, and yields of both ethanol and lactic acids (*R* ^2^ < 0.4 for all). However, some significant findings were obtained for the extreme cases. The lag time of substrate utilization for particle size <0.125 (Figure 1) was the longest, which was probably caused by the gelling observed for this particle size range, which created mass transfer limitations. On the other end of the spectrum tested, for particle size 2–4 mm, DP4+ production was significantly higher (Figure 1a) compared to the other size ranges, as confirmed by ANOVA and Tukey's test. The maximum glucose produced (Figure 1b) was also the lowest for this particle size. This is expected since starch hydrolysis due to amylase activity should yield less glucose and more high molecular weight products (DP4+) as the particle size increases. Sugar recovery percentages given in Table 1 support this claim, they decrease from 72% to 42% as the particle size increases.

### 3.2 Effect of initial loading rate

Based on the results obtained in the first part, two particle size ranges, one small and one large were selected for the loading rate study. Here, the gelling effect observed in <0.125 particle size range became even worse and rendered the media highly viscous for higher PPW loading rates, so it was discarded as an option and the next size range (0.125–0.250 mm) was chosen as the small size. For the large size, 1–2 mm range was chosen considering the unusual DP4+ production observed for the larger size.

A higher PPW loading rate means more PPW and consequently, more starch and carbon sources are available for *R. oryzae* to produce lactic acid and ethanol. Therefore, concentrations of lactic acid and ethanol are expected to be dependent on the PPW loading rate. In order to test this, in addition to 2% PPW loading rate used in the previous section, two more rates (4% and 8%) were tested on small (0.125–0.250 mm) and large (1–2 mm) PPW sizes. Due to high viscosity, PPW suspensions with a loading rate above 8% (w/v) could not be appropriately mixed and were, therefore, not investigated.

**FIGURE 2:**
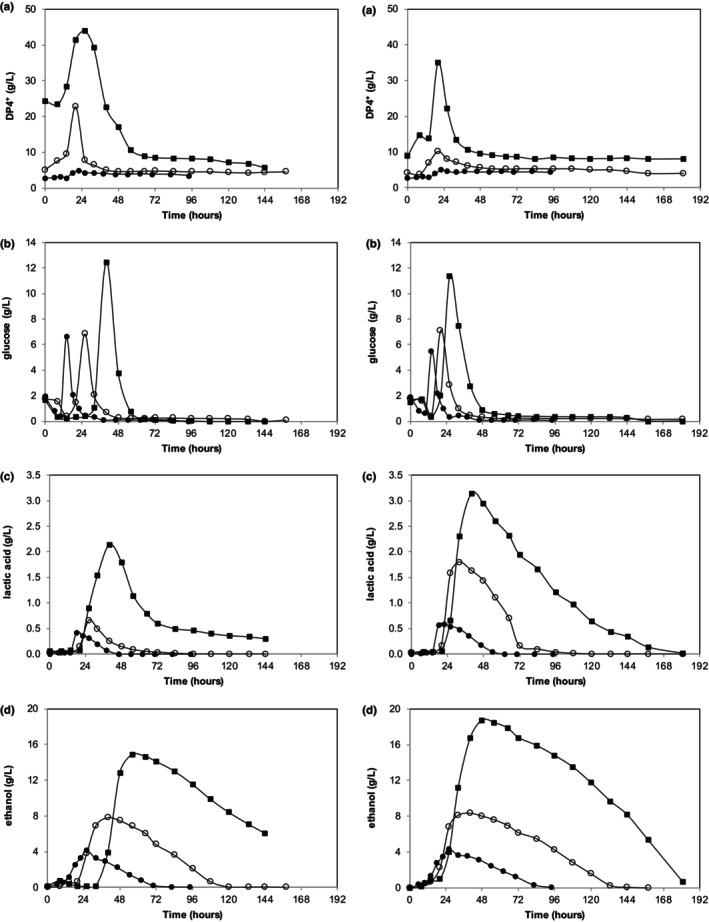
Time‐dependent concentration profiles of measured compounds (DP4+ (a), glucose (b), lactic acid (c), and ethanol (d)) obtained under different PPW loading rates (●: 2, ○: 4, ■: 8%). Left: small (0.125–0.250 mm) and Right: large (1–2 mm) PPW sizes.

Time‐dependent concentration profiles of measured compounds (DP4+, glucose, lactic acid, and ethanol) obtained under different PPW loading rates (2%, 4%, 8%) for both small and large particle sizes are given in Figure 2. Calculated bioprocess performance data (sugar recovery, substrate conversion efficiency, maximum amounts produced, average production rates, and yields) are tabulated in Table 2.

**TABLE 2:** Effect of the PPW loading rate on the performance of the fermentations.

PPW loading rate (%)	PPW particle size (mm)	Sugar recovery (%)	Substrate conversion efficiency (%)	Ethanol				Lactic acid			
				Time of peak production (h)	Maximum amount produced (g/L)	Average production rate (g/L day)	Yield (g/g_PPW_)	Time of peak production (h)	Maximum amount produced (g/L)	Average production rate (g/L day)	Yield (g/g_PPW_)
2	0.125–0.250	66.0 ± 2.0	43.1 ± 1.5	26	4.19 ± 0.14	3.87 ± 0.13	0.209 ± 0.007	18	0.42 ± 0.04	0.56 ± 0.05	0.021 ± 0.002
4	0.125–0.250	33.9 ± 1.2	40.2 ± 0.5	40	7.88 ± 0.07	4.72 ± 0.04	0.197 ± 0.002	26	0.66 ± 0.06	0.61 ± 0.05	0.017 ± 0.001
8	0.125–0.250	30.9 ± 1.5	39.1 ± 0.7	56	14.90 ± 0.36	6.39 ± 0.15	0.186 ± 0.005	40	2.14 ± 0.18	1.28 ± 0.11	0.027 ± 0.002
2	1.000–2.000	54.7 ± 2.4	45.6 ± 2.0	26	4.36 ± 0.22	4.03 ± 0.20	0.218 ± 0.011	22	0.58 ± 0.04	0.63 ± 0.04	0.029 ± 0.002
4	1.000–2.000	36.2 ± 2.1	45.4 ± 0.8	40	8.36 ± 0.12	5.01 ± 0.07	0.209 ± 0.003	32	1.80 ± 0.10	1.35 ± 0.07	0.045 ± 0.002
8	1.000–2.000	28.0 ± 2.2	50.0 ± 1.4	48	18.83 ± 0.50	9.41 ± 0.25	0.235 ± 0.006	40	3.14 ± 0.16	1.89 ± 0.09	0.039 ± 0.002

Figure 2 shows that DP4+ and free glucose concentrations were dependent on the PPW loading rate applied (Figure 2a,b), a higher loading rate resulted in more DP4+ and glucose for both small and large particle sizes. Lag time for production was not affected by the loading rate for DP4+; however, it was considerably increased for the glucose as the loading rate increased.

It is also obvious from Figure 2c,d) that the PPW loading rate significantly affected ethanol and lactic acid production. Increasing PPW loading rates clearly increased the produced amounts, average production rates, and yields of both ethanol and lactic acids, for both small and large particle sizes (Table 2). Table 2 also shows that sugar recovery was also dependent on the loading rate; it decreased with the increasing loading rate, for both particle sizes tested.

Moreover, regression analysis revealed that produced lactic acid and ethanol amounts increased linearly with increasing the PPW loading rate (*R* ^2^ = .98 (lactic acid small size), *R* ^2^ = .97 (lactic acid large size), *R* ^2^ = .99 (ethanol small size), *R* ^2^ = .99 (ethanol large size)). This is expected, as the high loading rate means more substrate available to the cells which in turn should result in more product obtained. Similarly, increasing the PPW loading rate increased ethanol and lactic acid production rates. Again, the correlation can be described with a linear regression with an offset (*R* ^2^ = .99 (lactic acid small size), *R* ^2^ = .93 (lactic acid large size), *R* ^2^ = .99 (ethanol small size), *R* ^2^ = .98 (ethanol large size)). This is also expected, as the high loading rate may alleviate any substrate limiting step on the bioprocess rate. On the other hand, lactic acid and ethanol yields do not seem to depend on the PPW loading rate, with regression analysis giving mixed results and poor fits. By definition, the yield is based on amount produced per substrate, so increasing substrate does not necessarily increase product conversion efficiency.

Lastly, it can be deduced from Figures 2c,d that higher PPW loading rates caused slightly longer lag times for both ethanol and lactic acid production.

## 4 DISCUSSION

Since the fermentation was carried out in unsteady state batch mode, the rates of production and consumption changed during the runs. In such cases, the maximum rate or average rate can be calculated and utilized for analysis. In practice, the instantaneous maximum rate achieved is impossible to obtain for the entire process duration. Therefore, in our case, average rates were calculated and are given in Tables 1 and 2, which in our opinion, reflect the overall performance better.

The peak amount obtained and duration until that peak time was utilized to calculate the average production rates.

Tables 1 and 2 also report yields and sugar recovery. Here, since products were utilized back by the microorganism, the peak amount obtained and duration until that peak time was utilized to calculate the yields, based on the assumption that the batches would be stopped at those times in an industrial process. Sugar recovery shows the efficiency of the microorganism to convert the starch available in the PPW to glucose.

Substrate conversion efficiency is another particularly useful parameter for characterizing ethanol and lactic acid production and is reported in Tables 1 and 2.

Figures 1 and 2 show that glucose, DP4+, ethanol, and lactic acid concentrations peak at 24 h (8% loading rate slightly retards the peak). This is interesting because the metabolism of filamentous fungi is slower compared to bacteria or yeast (Alsuhaim et al., 2012; Sahu et al., 2015).

The fast process is probably due to the species used, *Rhizopus* spp. grow faster than other fungi (Meletiadis et al., 2001). Process conditions used also affect the metabolic rate, filamentous fungi were shown to grow faster in liquid media compared to solid media (Kosegarten et al., 2017). Indeed, according to prior research (Fan et al., 2022; Ozer Uyar et al., 2016), *R. oryzae* reaches the stationary phase after 18–24 h, depending on pH, temperature, the working volume of the growth medium, and the initial spore concentration. In the log phase of growth primary metabolites and enzymes are synthesized, one of which is amylase, therefore DP4+ and glucose concentrations peak at this time. Lactic acid and ethanol production follow closely.

Even though there is no other published study previously in the literature that explores the use of PPW by *R. oryzae* as thoroughly as this study, some comparable works exist that either report utilization of other agro‐wastes by *R. oryzae* or the utilization of PPW by other microorganisms. The results of those trials are summarized in Table 3.

**TABLE 3:** Fermentation of agro‐wastes to obtain lactic acid and ethanol.

Strain	Type	Substrate		Lactic acid production			Ethanol production			Refs.
		Pretreatment (hydrolysis)	Particle size (PS), loading rate (LR)	Conc. (g/L)	Rate (g/L day)	Yield (g/g)	Conc. (g/L)	Rate (g/L day)	Yield (g/g)	
*R. oryzae*	PPW	None	1–2 mm PS, 8% LR	3.1	1.9	0.04	18.8	9.4	0.24	This work
*R. oryzae*	PPW	Heat & acid	1.2–1.6 PS	66.5	–	–	–	–	–	Kumar and Shivakumar (2014)
*S. cerevisiae*	PPW	Heat & enzyme	0.2 mm PS, 10%–15% LR	–	–	–	0.25–0.4	–	–	Meenakshi and Kumaresan (2014)
*S. cerevisiae*	PPW^h^	Acid & enzyme	2% LR	–	–	–	4.2–7.6	4.0	0.46	Arapoglou et al. (2010)
*S. cerevisiae*	PPW	Heat & acid & enz.	12.5% LR	–	–	–	11.5	32	0.15	Chohan et al. (2020)
Mixed culture	PPW	None	3%–5% LR	11	125 mg/g day	0.25	–	–	–	Liang et al. (2015)
Mixed culture	PPW^h^	Heat & enzyme	2.5% LR	0.2–5.1	–	0.01–0.18	–	–	–	Breton Toral et al. (2016)^a^
Mixed culture	PPW	Heat & enzyme	2% LR	5.3	–	0.22	1.1	–	–	Liang et al. (2014)^b^
*R. oryzae*	Agro‐food wastes^e^ ^,^ ^h^	Heat & acid	10% LR	69–79	–	–	–	–	–	Ranjit and Srividya (2016)
*R. oryzae*	Agro‐food wastes^f^ ^,^ ^h^	Heat & acid	4% LR	34–70	–	–	–	–	–	Mudaliyar and Kulkarni (2012)
*R. oryzae*	Waste paper^h^	Enzyme		49	16.3	0.59	–	–	–	Park et al. (2004)
*R. oryzae*	Pineapple waste	None	0.5–1 mm PS	104 mg/g	10 mg/g day	0.00045	–	–	0.00014	Zain et al. (2021)^d^
*R. oryzae*	Agro‐food wastes^g^	Heat & acid	1.2–1.6 mm PS	67–72	24	3.5–3.6	–	–	–	Kumar and Shivakumar (2014)
*R. oryzae*	Cassava pulp^h^	Acid pretreated	7% LR	6.7	3.8	0.09	16.8	14.9	0.35	Thongchul et al. (2010)
*R. oryzae*	Cassava pulp^h^	Enzyme	7% LR	16.7	7	0.24	29.2	9.8	0.48	Thongchul et al. (2010)
*R. oryzae*	*Sophora flavescens*	Alkali & enzyme	10% LR	30.6	5.5	–	0.7	–	–	Ma et al. (2020)^c^
*R. oryzae*	Wheat wastewater	None	10%–100% wastewater	0–5.6	–	–	–	–	–	Göçeri et al. (2021)
*R. oryzae*	Yam peel^h^	Heat & acid	8% sugar LR	50.5	–	0.75	–	–	–	Ajala et al. (2021)

^a^ Acetic acid production was also reported: 0.2–0.4 g/L.

^b^ Acetic acid production was also reported: 1.4 g/L.

^c^ Acetic acid production was also reported: 2.6 g/L.

^d^ Solid state fermentation.

^e^ Different agro‐food wastes were used: rice, wheat, ragi bran, rice starch water, tea waste, sugar cane bagasse, groundnut, and coconut oil cakes.

^f^ Different agro‐food wastes were used: dry grass, coconut husk, sugarcane waste, and wood chips.

^g^ Different agro‐food wastes were used: sapota, banana, papaya peel, corn cob powder, and carboxymethyl cellulose.

^h^ Hydrolysates of the wastes were used.

The only comparable study in the literature involved drying, powdering, and sieving PPW similar to this work; however, only a single particle size (1.2–1.6 mm) was used. Obtained PPW was pretreated by steam explosion and acid hydrolysis before fermentation by *R. oryzae*. However, the authors reported very limited results for this process (only the final lactic acid concentration was given as 66.5 g/L) (Kumar & Shivakumar, 2014).

Studies that utilized PPW employed yeast (*S. cerevisiae*) (Arapoglou et al., 2010; Chohan et al., 2020; Meenakshi & Kumaresan, 2014) or mixed (bacterial or unidentified) cultures (Breton Toral et al., 2016; Liang et al., 2014, 2015) for fermentation and reported lactic acid and ethanol production amounts in the range of 0.2–11 and 0.3–11.5 g/L, which are comparable to this study (3.1 and 18.8 g/L).

Studies that used *R. oryzae* deal with the utilization of agro‐food waste materials (i.e., peels, brans, husks) as substrates for lactic acid and ethanol production. Some studies compared different substrates (Kumar & Shivakumar, 2014; Mudaliyar & Kulkarni, 2012; Ranjit & Srividya, 2016), whereas others focused on the utilization of a single substrate (Ajala et al., 2021; Göçeri et al., 2021; Ma et al., 2020; Park et al., 2004; Thongchul et al., 2010; Zain et al., 2021). Lactic acid was produced in all cases; however, ethanol production was not always reported. Acetic acid was also produced in some cases (Breton Toral et al., 2016; Liang et al., 2014; Ma et al., 2020). Concentration, yield, and rate values covered a wide range due to different substrate and operating conditions but it can be deduced from Table 3 that ethanol production performance in this study was on par with other studies, whereas lactic acid production performance was poorer.

This can be attributed to the substrate pretreatment; most of the studies used pretreatments (heat, enzyme, acid) for hydrolysis of the substrate prior to the fermentation step, which contrasts this work in which PPW substrate was utilized as is. Studies that explored effect of pretreatments also confirmed the improvement in bioprocess performance (Breton Toral et al., 2016; Liang et al., 2014).

Some studies used filtered hydrolysates obtained from wastes instead of using solid waste particles directly (Ajala et al., 2021; Park et al., 2004; Thongchul et al., 2010), which obviously enhances production due to improved mass transfer and reduced viscosity.

However, it should also be noted that such pretreatments have a cost (consumables, equipment, time), and for low‐value substrates such as PPW, that additional cost of pretreatments may not justify increased yield and rate. Techno‐economic analysis would be required for larger scale operations to assess and validate the requirement of pretreatments for PPW utilization.

It is interesting that usually only lactic acid production was reported, even though *R. oryzae* possesses lactate dehydrogenase, pyruvate decarboxylase, and alcohol dehydrogenase enzymes for the coproduction of ethanol and lactic acid. It is previously documented that both metabolites are produced in defined media by *R. oryzae*, high spore inoculation shifts production from lactic acid to ethanol, and their yields increase as the initial glucose amounts increase (Büyükkileci et al., 2006).

Future research directions and recommendations drawn from the key findings of this study are listed below.

Gelling was observed in the nutrient media prepared with PPW particle sizes smaller than 0.125 mm, and increased media viscosity resulted in long lag times for production.

*R. oryzae* can utilize PPW as a substrate to produce DP4+, glucose, lactic acid, and ethanol. However, it consumes those metabolites back during the fermentation, glucose, and DP4+ were utilized first, and then lactic acid and ethanol were used. Lactic acid and ethanol concentrations should be monitored as the bioprocess parameters and the batch should be stopped when their concentrations peak.

Recommended parameters are PPW particle size of 1–2 mm and loading rate of 8%; the highest maximum amounts of ethanol (18.83 g/L) and lactic acid (3.14 g/L), highest rates of ethanol (9.41 mg/L day) and lactic acid production (1.89 g/L day), the highest yield of ethanol (0.235 mg/g PPW), and second highest yield of lactic acid (0.039 mg/g PPW) were obtained under these conditions.

## AUTHOR CONTRIBUTIONS

**Gülsüm Ebru Ozer Uyar:** Conceptualization (lead); data curation (lead); formal analysis (supporting); funding acquisition (lead); methodology (equal); resources (lead); writing – original draft (supporting); writing – review and editing (equal). **Basar Uyar:** Formal analysis (lead); investigation (supporting); methodology (equal); visualization (lead); writing – original draft (lead); writing – review and editing (equal).

## FUNDING INFORMATION

This research was funded by Kocaeli University BAP funds, grant number FYL‐2020‐2162.

## CONFLICT OF INTEREST STATEMENT

The authors declare no conflict of interest. The funders had no role in the design of the study; in the collection, analyses, or interpretation of data; in the writing of the manuscript, or in the decision to publish the results.

## Data Availability

The data that support the findings of this study are available from the corresponding author, upon reasonable request.
